# Endocrine and dog factors associated with semen quality

**DOI:** 10.1038/s41598-024-51242-0

**Published:** 2024-01-06

**Authors:** Ida Hallberg, Hannah Olsson, Angus Lau, Stina Wallander, Anna Snell, Daniel Bergman, Bodil Ström Holst

**Affiliations:** 1 Department of Clinical Sciences, Division of Reproduction, The Centre for Reproductive Biology in Uppsala, Swedish University of Agricultural Sciences, 750 07 Uppsala, Sweden; 2 Department of Biomedical Sciences and Veterinary Public Health, Swedish University of Agricultural Sciences, 750 07 Uppsala, Sweden; 3 Department of Laboratory Medicine, Karolinska Institute, 141 86 Stockholm, Sweden

**Keywords:** Biomarkers, Endocrinology, Urology

## Abstract

Knowledge of factors associated with semen quality may help in investigations of the aetiology and pathophysiology. We investigated the correlation between biomarkers for testicular cell function (anti-müllerian hormone, AMH, Inhibin B, testosterone, free androgen-index (testosterone/sex-hormone binding globulin), insulin like peptide 3, INSL-3), alkaline phosphate (ALP), canine prostate-specific esterase (CPSE), and heterophilic antibodies with dog variables, semen quality, and fertility. Blood and semen were collected from 65 Bernese Mountain Dogs. We evaluated total sperm count, motility and morphological parameters. The semen quality ranged from poor to excellent, with an average total sperm count of 1.1 × 10^9^ and 50% morphologically normal spermatozoa (MNS). Age and abnormal testicular consistency correlated with decreased motility and MNS. Higher ALP correlated with higher total sperm count. AMH could not be detected in seminal plasma. AMH in blood correlated with head defects and high AMH concentration correlated with a severe decline in several semen parameters. Testosterone was negatively and CPSE positively correlated with age. No correlations were found for INSL-3, inhibin B, or heterophilic antibodies. Our findings contribute to the understanding of factors associated with semen quality in dogs, particularly related to Sertoli cell function.

## Introduction

Infertility is a prevailing concern within the canine population. While reproductive success is essential for maintaining the health and genetic diversity of the species, the occurrence of infertility poses challenges for breeding programs, particularly when considering the impact of neutering or spaying on breeding potential at a population level. Certain breeds have been observed to be more affected with reproductive impairments, such as decreased semen quality or compromised fertility rates (e.g.^1,2^). These breed-specific differences highlight the importance of investigating the underlying factors related to infertility and implementing targeted interventions tailored to specific breeds.

Infertility may be attributed either to the bitch, the stud dog or factors affecting both partners. While the cause of infertility may be challenging to address in the bitch, the semen quality poses a biological marker for fertility in the male. A male dog presenting at the veterinary clinic with a history of failure to achieve pregnancy will typically be subjected to a full andrological examination including a semen sample. Ejaculate volume, colour, sperm concentration and morphology are recorded and typically evaluated as normal when the proportion motile spermatozoa reaches > 70% and > 60% of the spermatozoa display normal morphology^3,4^. If indicated extended analyses such as cytology, bacteriology or concentration of alkaline phosphate (ALP) in the seminal plasma is assessed.

Spermatozoa develop in the testes, where the Leydig and the Sertoli cells provide structural and nutritional support in addition to producing hormones and paracrine factors essential for spermatogenesis. Biomarkers produced by the Leydig or the Sertoli cell may thus provide information on pathogenesis of specific defects of spermatozoa. Testosterone is produced by the Leydig cell in response to the secretion of luteinizing hormone (LH) and is responsible to the development of male characteristics. The concentration of testosterone is lower in men with inferior sperm quality and decreases with age in both men and male dogs^5,6^. In the circulation, testosterone is largely protein-bound to albumin and more specifically sex-hormone binding globulin, SHBG, and only the free fraction of testosterone may exert biological effects^7^. Insulin-like peptide 3 (INSL-3) is also produced by the Leydig cell but rather reflects the chronic status of the hypothalamic-pituitary–gonadal axis^8^.

Anti-Müllerian hormone (AMH) is secreted by Sertoli cells. Because of the expression of AMH ligand and receptor during spermatogenesis and follicular recruitment, is has been hypothesised that the AMH function is involved in the germ cell maturation in both males and females^9^. A moderate negative association between AMH and semen quality, especially decreased motility and the proportion morphologically normal spermatozoa, has been described in dogs^10^, and the serum AMH concentration is increased in dogs with Sertoli cell tumours^11–13^. Inhibin B is another hormone produced by the Sertoli cell. With functions involved in the regulation of steroidogenesis, it has shown potential for predicting semen quality in some species^14^.

The aetiology of decreased semen quality in dogs is multifactorial and include fever/infections and prostatic disease. Subfertility in the male dog may be counteracted with optimal breeding management^15^. Even so, the prognosis for a dog presenting with poor semen quality is often poor^15^. In dogs with non-obstructive azoospermia, immune-mediated orchitis has been suggested as a common cause, and the presence of anti-thyroidal antibodies suggested a possible association with thyroid disease in some dogs^16^. Rheumatoid factor in autoimmune disease in humans act as heterophilic antibodies and may cause assay interference^17^. Heterophilic antibodies are widespread in the canine population in general^18^ and in Bernese mountain dogs in particular^19^, and their possible association with autoimmune orchitis is unknown. Although much research on factors that are correlated with semen quality has been conducted in other species, there are fewer studies in dogs. For example, decreased semen quality with increasing age is well established in humans^20^. In aging dogs, the proportion of spermatozoa with proximal droplet increase whereas the effect of age on other semen parameters such as percentage motile spermatozoa and total sperm count is less clear^21,22^.

In the present study^7,14^, we investigated dog factors and endocrine variables, and their potential association to semen quality and fertility, to gain more knowledge on aetiology and pathophysiology of sperm defects and semen quality in the dog. We analysed a range of biomarkers produced by the Leydig (Testosterone, Free Androgen Index: FAI, INSL-3) and the Sertoli cell (AMH, Inhibin B). In addition, a marker for prostatic size, canine prostate specific esterase (CPSE), was included, as was the presence of heterophilic antibodies and ALP. To reduce the variation caused by e.g. dog size^22^, we limited our investigation to include one dog breed, the Bernese mountain dog.

## Results

### Dog characteristics and semen quality

Dog characteristics are presented in Table 1. Dogs were on average 4.5 years old (mean 53 months, range 16–137) with a mean weight 50 kg (range 35–58). The body condition score (BCS) of the dogs was normal to slightly overweight (BCS 5, 3–7).

**Table 1:** Dog characteristics, semen quality and serum concentration of biomarkers for testicular cell function in the study population.

Variable	n	Value (dispersion)
Age [months], mean (SD)	65	53.3 (3.8)
Weight [kg], mean (SD)	65	49.4 (0.6)
BCS [scale 1–9], median (range)	65	5 (3–7)
Treatment with antiparasitic drugs [number] (%)	65	48 (74%)
Testicle diameter [cm], mean (SD)	65	55.5 (1.6)
Motile sperm [%] mean (SD)	62	63.9 (3.1)
Total sperm count [× 10^9^] mean (SD)	64	1.04 (0.10)
Morphologically normal sperm (MNS) [%], mean (SD)	62	49.8 (3.1)
Tail defects^a^ [%], mean (SD)	62	24.3 (2.4)
Pathological heads [%], mean (SD)	62	6.8 (0.7)
Midpiece defects [%], mean (SD)	62	3.1 (0.4)
Proximal droplets [%], mean (SD)	62	13.4 (1.8)
Alkaline phosphate, ALP, in seminal plasma [× 1000 U/l], median (IQR)	64	11.1 (3.9–19.7)
Anti-müllerian hormone, AMH [ng/mL], median (IQR)	64	5.0 (3.7–6.8)
Total testosterone [nmol/l], median (IQR)	64	7.1 (4.6–10.3)
Free androgen index, FAI^b^, median (IQR)	64	0.11 (0.05–0.21)
Canine prostate-specific esterase, CPSE [ng/ml], median (IQR)	64	500 (152–500)
Insulin-like Peptide 3, INSL-3 [pg/ml] median (IQR)	64	91.0 (5.2–163)
Inhibin [ng/l], median (IQR)	63	13.7 (9.2–17.8)

^a^Sum of single bent tails, coiled tails and double bent tails.

^b^Calculated from total testosterone / SHBG.

The proportion of dogs with normal testicles by palpation was 63%, the remaining 37% had one or two testicles considered abnormal by palpation. The diameter of both testicles (scrotal width) was on average 5.6 cm (standard deviation ± 1.27). The coefficients of variation (CV) upon 2–3 persons independently measuring the testes were on average 16.3% (range 1.2–34) for estimated testicular volume and 7.6% (0–18) for testicle diameter. When one person measured the testes repeatedly, the CV for the volume was 9.5% (5–17) and 5.2% (2–9) for the diameter (details in Table S2a-b).

Semen samples were obtained from 65 of the 67 dogs. One azoospermic sample was excluded due to an incomplete ejaculate or obstructive azoospermia based on a low concentration of ALP, and 64 samples were evaluated for total sperm count. Samples with secretory azoospermia (n = 2) were excluded from morphological assessment resulting in 62 samples evaluated for morphological parameters.

The semen quality of the dogs ranged from very poor to excellent (Table 1). The mean total sperm count was 1.1 × 10^9^ (0.0–5.7). The mean proportion of morphologically normal sperm (MNS) was 50% and the most prominent sperm defects observed were tail defects (mean 26.5% of the sperm) and proximal droplets (14.1%). The vast majority (80%) of the dogs had > 300 million spermatozoa in the ejaculate, and about half had a motility > 70%. The proportion of MNS ranged from 10 to 88% and 34% had a proportion of MNS > 60%. The total number of MNS in the ejaculate was on average 522 million (range 0–1790 × 10^6^).

### Semen quality and fertility

Semen quality in dogs with previous matings and therefore known recent fertility were investigated. Previous matings (any time) were reported for 44 of the 65 dogs and 80% of these were reported to have produced offspring. Recent mating were reported in 19 dogs. Of this subset of matings, 15 (74%) resulted in a confirmed pregnancy. Semen quality in dogs with recent mating and a confirmed pregnancy and dogs with no resulting pregnancy is described in Table 2. Among the successful pregnancies was one dog with 18.5% MNS. This dog presented with a high number of tail defects and a high proportion of proximal droplets but with normal sperm count and motility. There was no significant difference in semen quality between dogs with and without successful pregnancy in this small sample set.

**Table 2:** Dog characteristics and semen quality in 19 dogs that mated two months before or after semen quality analyses.

Variable	No pregnancy (n = 5)	Pregnancy (n = 14)
Age, [months], mean (SD)	52 (12.9)	39 (6.1)
BCS, [scale 1–9], median (IQR)	5 (5–6.5)	5 (4–5)
Testicle diameter, [cm], mean (SD)	5 (1.9)	5.3 (3.1)
Total sperm count, [× 10^9^] mean (IQR)	1.15 (0.51–1.39)	1.18 (0.68–1.42)
Motility, [%] (IQR)	55 (25–77)	80 (70–98)
MNS, [%] (IQR)	44 (15–69)	60 (42–79)
Tail defects, [%] (IQR)	14 (2.5–50.3)	13.1 (5.8–33)
Head defects, [%] (IQR)	12.3 (3.9–20.5)	8.6 (6.9–15.2)
Midpiece defects, [%] (IQR)	2.5 (1–2.8)	2.8 (1.7–4.9)
Proximal droplets, [%] (IQR)	4 (1.25–20.8)	8 (5.2–12.1)
Good semen quality^§^ [n] (%)	1 (20%)	5 (35%)

Data presented as median (interquartile range, IQR) or mean (standard deviation, SD), Good semen quality defined as morphologically normal spermatozoa, MNS ≥ 60% and Motility ≥ 70% and total sperm count ≥ 300 million spermatozoa ^3,25^, presented as number and percentage of the dogs in categories “no pregnancy” and “pregnancy”.

### Analytes in serum and seminal plasma

The results for the quantified analytes are presented in Table 1. Median ALP was 11 000 U/l (3900–19 700) and showed a great variation in ejaculates containing spermatozoa with values from 5 U/l to close to 4 × 10^6^ U/l. Anti-müllerian hormone (AMH) was not detected (< 0.2 ng/mL) in seminal plasma from any dog (for details, see Table S3, SI). Median AMH in serum was 5.1 ng/ml (3.7–6.8). Five dogs had an AMH concentration above 10 ng/mL. Median total testosterone concentration was 7.1 nmol/l (4.6–10-3) ranging from < LOD to 50.1 nmol/l. All dogs with testosterone below the detection limit (n = 4) had poor total sperm count or very low MNS while the dog with the highest concentration had excellent semen quality. More than half of the dogs (55%) had a canine prostate-specific esterase (CPSE) concentration above the measurable range, > 500 ng/ml. Eighty five per cent of the dogs had a concentration of CPSE > 90 ng/ml.

### Correlation between dog variables and semen quality

Dog variables such as age, BCS and testicular status was correlated with semen quality in the included dogs. In our population, 16 months to 11 years old, a decreased motility (*p* = 0.01) and proportion of MNS (*p* = 0.02), was observed with increasing age (Table 3). The average motility decreased with 28% and the proportion MNS with 20% from dogs < 3 years of age to dogs > 6 years of age. The decrease in MNS was driven by an increased proportion of proximal droplets (*p* = 0.01). Testicle consistency correlated with semen quality. Abnormal testicular consistency was associated with decreased motility (*p* = 0.001) and MNS (*p* = 0.001) driven by increased proportion of proximal droplets (*p* = 0.01, Table 4). There was also a tendency for decreased total sperm count (*p* = 0.09) and increased head defects (*p* = 0.07) with abnormal testicle consistency. We could observe a significantly decreased proportion of tail defects (*p* = 0.04) with increasing BCS, but no other significant correlation. There was no significant correlation between testicle diameter and semen quality or dog variables (Table 3).

**Table 3:** Spearman’s correlation between dog variables and semen parameters.

Variable	Age		BCS		Testicle diameter	
	ρ (CI)^a^	*p*	ρ (CI)^a^	*p*	ρ (CI)^a^	*p*
Motility	− **0.32(**− **0.53;0.07)**	0.01*	0.16(− 0.10;0.40)	0.23	0.07(− 0.18;0.32)	0.57
Total sperm count	− 0.17 (− 0.40;0.08)	0.17	0.01 (− 0.23;0.26)	0.90	0.09(− 0.16;0.33)	0.47
MNS	− **0.30 (**− **0.51;0.05)**	0.02*	0.04 (− 0.23 − 0.28)	0.78	0.06(− 0.19;0.30)	0.67
Head defects	0.18 (− 0.07;0.41)	0.15	0.17 (− 0.08;0.40)	0.18	0.04(− 0.20;0.29)	0.73
Midpiece defects	0.09 (− 0.16;0.32)	0.50	0.10 (− 0.15;0.33)	0.50	0.08(− 0.17;0.32)	0.55
Tail defects	− 0.07 (− 0.30;0.18)	0.58	− **0.25(**− **0.47;0.01)**	0.04*	0.02(− 0.22;0.26)	0.87
Proximal droplets	**0.31 (0.07;0.52)**	0.01*	0.08 (− 0.17;0.32)	0.51	0.01(− 0.23;0.25)	0.91

Definition of tails, heads, FAI, dataset for total sperm count and morphology.

^a^Spearman’s correlation (95% confidence interval). Bold text and asterisk indicate significant (*p* < 0.05) correlations.

**Table 4:** Testicular consistency and Canine prostate-specific esterase, CPSE, in relation to semen quality and age.

Variable	Canine prostate-specific esterase, CPSE			Testicular consistency		
	> 90 ng/ml, n = 55	< 90 ng/ml, n = 10	*p*-value	Normal, n = 40	Abnormal, n = 23	*p*-value
Age, [months] mean (SD)	**67.1 (7.3)**	**20.5 (3.5)**	< 0.0001*	46.2 (4.1)	63.1 (7.2)	0.06
Motility, [%] (SD)	50.2 (6.1)	55 (25)	0.08	**71.2 (3.1)**	**50.7 (5.8)**	0.001*
Total sperm count, [× 10^9^] mean (SD)	0.78 (0.14)	1.3 (1.2)	0.86	1.1 (0.1)	0.81 (0.2)	0.09
MNS [%]	37.8 (5.1)	14.3 (4.3)	0.47	**58.6 (3.4)**	**35.7 (4.9)**	0.001*
Head defects [%]	8.1 (1.5)	26.6 (11.7)	0.53	5.5 (0.4)	9.8 (1.9)	0.07
Midpiece defects [%]	2.7 (0.4)	18.9 (7.4)	0.62	2.7 (0.3)	4.1 (1.1)	0.54
Tail defects [%]	27.0 (4.0)	15.0 (10.0)	0.87	24.4 (3.4)	25.9 (3.7)	0.52
Proximal droplets [%]	16.8 (3.4)	33.8 (2.5)	0.89	**9.8 (1.5)**	**18.3 (3.3)**	0.01*

Testicular consistency assessed by manual palpation, considered abnormal if one or two testicles were abnormal by palpation. CPSE considered high if > 90 ng/ml corresponding to a prostate that is 2.5 times larger than that of a 4-year-old dog of the same weight, with increased risk of developing clinical signs^51^. Difference between group tested with Kruskal Wallis, *p* < 0.05 considered significant. Bold text and asterisk indicate significant (*p* < 0.05) correlations.

### Correlation between biomarkers and semen quality

To gain more knowledge on aetiology and pathophysiology of sperm defects and semen quality in the dog, we investigated endocrine variables, and their potential association to semen quality. Results are presented in Tables 4, 5. Higher ALP values correlated with higher total sperm count in ejaculates with spermatozoa (*p* < 0.001). In the subset of dogs with > 300 and > 600 million spermatozoa in the ejaculate, this correlation persisted (details in Table S4a-b, SI). In samples containing spermatozoa but with low ALP (defined with the cut-off of < 5000 IU^23,24^, n = 17), the average total sperm count (average 600 × 10^6^) was lower compared to in samples with ALP > 5000 IU (1.3 × 10^9^, *p* = 0.001). Likewise, in samples with low ALP, the concentration was lower (85 × 10^6^) compared to in samples with high ALP (184 × 10^6^, *p* = 005). Four samples with ALP < 5000 IU had > 10^9^ spermatozoa in the ejaculate. One sample with ALP 354 IU had 1.8 × 10^9^ spermatozoa and overall good semen quality.

**Table 5:** Correlation between biomarkers and semen parameters.

	ALP		AMH		Tot testosteron		FAI		Inhibin		INSL-3	
	ρ (CI)^a^	*p*	ρ (CI)^a^	*p*	ρ (CI)^a^	*p*	ρ (CI)^a^	*p*	ρ (CI)^a^	*p*	ρ (CI)^a^	*p*
Age	− 0.16 (− 0.40;0.09)	0.21	0.08 (− 0.18;0.31)	0.61	− **0.27(**− **0.49;** − **0.01)**	0.03*	− **0.35 (**− **0.56;** − **0.10)**	0.01*	− 0.16(− 0,40;0,09)	0.21	0.13 (− 0,12; 0,37)	0.30
Motility	0.13(− 0.13;0.37)	0.33	− 0.20(− 0.43;0.06)	0.12	0.13 (− 0.13;0.37)	0.21	0.20 (− 0.05;0.44)	0.12	0.16(− 0,10;0,40)	0.22	− 0.16 (− 0,40; 0,10)	0.23
Total sperm count	**0.50 (0.28;0.67)**	0.00*	− **0.26(**− **0.48;** − **0.01)**	0.04*	0.20 (− 0.05;0.43)	0.11	0.12 (− 0.13;0.36)	0.33	0.25 (− 0.00;0.47)	0.05	− 0.14 (− 0.37;0.11)	0.28
MNS	0 − 07 (− 0.18;0.32)	0.57	− 0.14(− 0.38;0.12)	0.29	0.16 (− 0.10;0.40)	0.21	0.20 (− 0.05;0.43)	0.12	0.15(− 0,11; 0,39)	0.26	− 0.04 (− 0,29; 0,21)	0.75
Head defects	− 0.18 (− 0.41;0.08)	0.17	**0.26(0.01;0.49)**	0.04*	− 0.03 (− 0.29;0.22)	0.82	− 0.06 (− 0.30;0.20)	0.66	− 0.08(− 0,32;0,18)	0.56	0.10 (− 0,16; 0,34)	0.47
Midpiece defects	0.10 (− 0.16;0.34)	0.44	0.15(− 0.10;0.39)	0.23	− 0.08 (− 0.32;0.18)	0.55	− 0.11 (− 0.35;0.14)	0.38	− 0.10(− 0,34;0,17)	0.49	0.18 (− 0,08; 0,41)	0.17
Tail defects	− 0.02 (− 0.27;0.23)	0.87	0.12(− 0.13;0.36)	0.34	− 0.14 (− 0.38;0.12)	0.28	− 0.04 (− 0.29;0.21)	0.77	− 0.06(− 0,31;0,19)	0.62	− 0.10 (− 0,34; 0,16)	0.45
Proximal droplets	− 0.13 (− 0.37;0.12)	0.30	− 0.06(− 0.31;0.19)	0.64	− 0.22 (− 0.44;0.04)	0.10	− 0.23 (− 0.46;0.02)	0.07	− 0.17(− 0,41;0,09)	0.19	0.20 (− 0,06; 0,43)	0.12

Definition of tails, heads, FAI, dataset for total sperm count and morphology.

^a^Spearman’s correlation (95% confidence interval), bold text and asterisk indicate significant (*p* < 0.05) correlations. *p* = 0.00 correspond to values < 0.0001.

**Figure 1:**
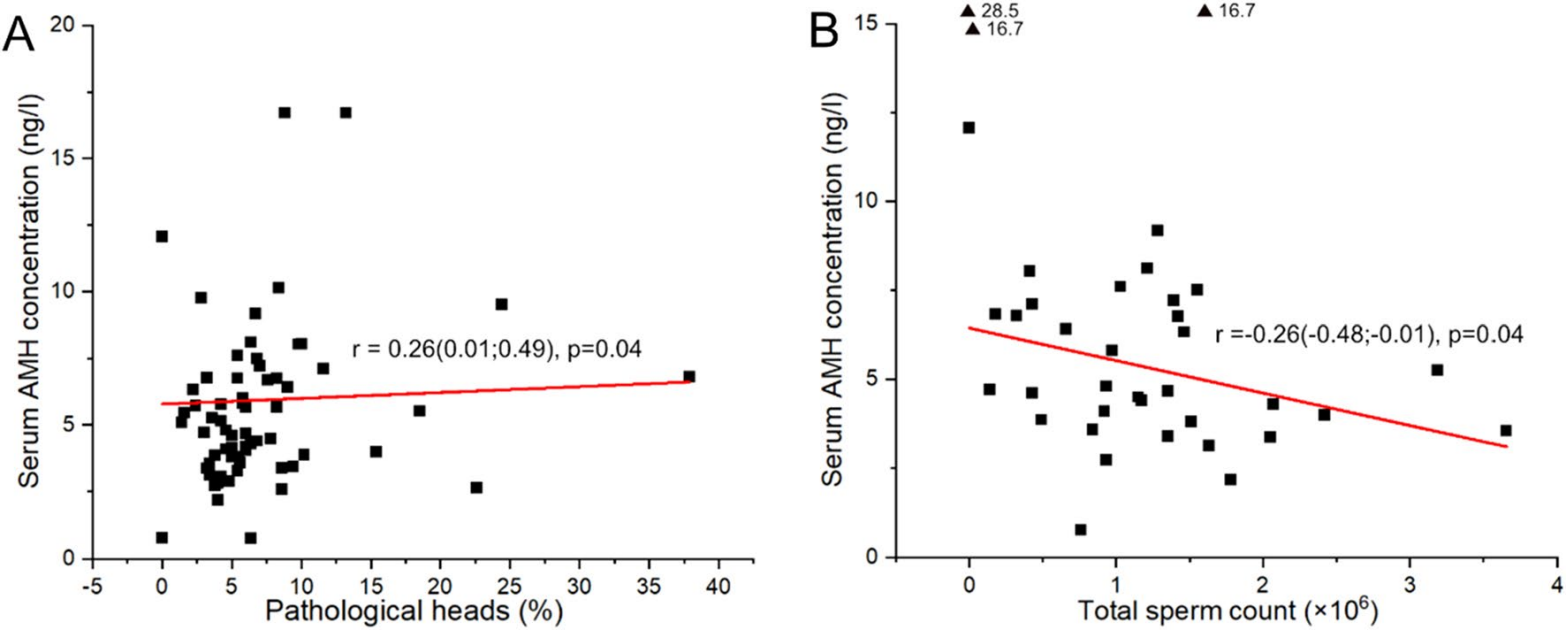
Association between serum AMH concentration and proportion pathological heads (**A**) and total sperm count (**B**). Values > 15 are depicted as triangles at the top of the graph. The red line visualizes the association between variables calculated using Spearman’s correlation, slope presented as r (confidence interval).

**Figure 2:**
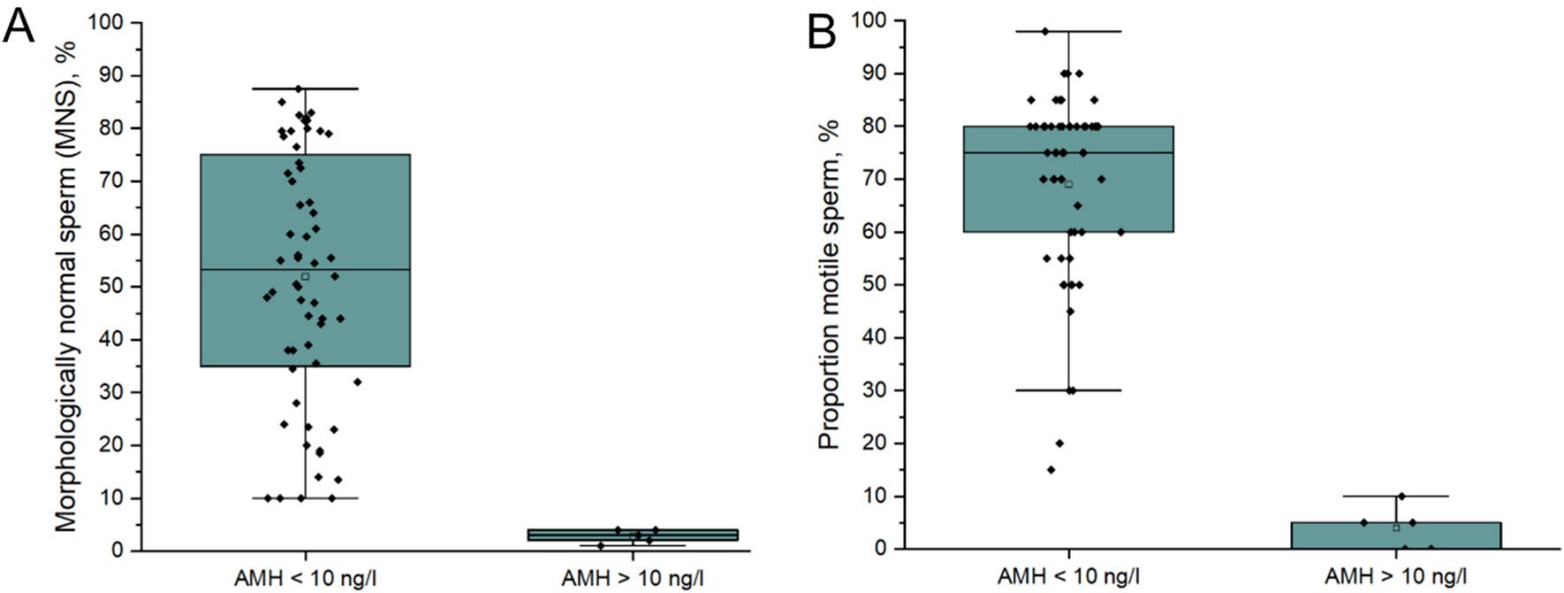
Serum AMH concentration in dogs < 10 ng/l and > 10 ng/l in relation to morphologically normal sperm, MNS (**A**) and proportion motile sperm (**B**). The data is visualized by individual value plots and a boxplot where the box correspond to data from the first to the third quartile (IQR), the bars represent 1.5 × IQR, the median is represented by a black line and the mean as a square with no fill.

An increasing concentration of serum AMH correlated with a decreasing total sperm count (*p* = 0.04) and an increasing proportion of sperm head defects (*p* = 0.04, Fig. 1). The correlation between AMH and semen parameters was driven by decreased semen quality in samples with high AMH (> 10 ng/ml) as illustrated in Fig. 2. AMH > 10 ng/ml was associated with increasing dog age (*p* = 0.003) and severely decreased motility (*p* < 0.0001) and MNS (*p* = 0.01). The median (IQR) sperm motility in dogs with > 10 ng/ml AMH in serum was 5% (0–7.5) compared to in dogs with AMH < 10 ng/ml, 75% (59–80). Similarly, the MNS count was 15% (11–26) in dogs with AMH > 10 ng/ml and 53% (35–76) in dogs with AMH < 10 ng/ml. There were too few observations to investigate specific defects in samples with AMH > 10 ng/mL. The sensitivity and specificity for AMH as a predictor of good semen quality (> 70% motility, > 60% MNS) was < 0.8, details can be found in Table S5, SI.

The total testosterone concentration decreased with age (*p* = 0.03) and was on average 8.6 ng/ml in dogs < 3 years of age and 6.4 in dogs > 6 years of age. There was a tendency toward an increased proportion of proximal droplets with decreasing testosterone levels (*p* = 0.10). Similarly, free androgen-index (FAI) correlated with dog age (*p* = 0.01) and decreasing FAI tended to correlate with increasing proportion of proximal droplets (*p* = 0.07). The CPSE concentration increased with dog age (*p* < 0.0001) and there was a tendency for lower motility with increasing CPSE (*p* = 0.08) but no correlation to other semen parameters. Insulin-like peptide 3 (INSL-3) and inhibin B were not correlated with dog age or semen quality, except for a tendency toward increased total sperm count with increasing inhibin B concentration (*p* = 0.05).

Heterophilic antibodies were detected in dogs (n = 6) with a semen quality ranging from poor to excellent (details in Table S6).

## Discussion

In the current study, we present parameters related to semen quality in dogs. The investigated variables included dog factors, such as age and factors assessed during routine clinical examination, and a wide range of biomarkers, including heterophilic antibodies and biomarkers representing testicular cell function and prostatic size. By including dogs of one specific breed, variation in semen quality related to size and weight^21,22^ could be decreased. The dogs were of varying age and semen quality ranged from excellent to very poor, creating good conditions for analyses of correlation between semen parameters and investigated factors.

The average semen quality of the 65 male Bernese mountain dogs was lower than what is considered normal for dogs^3^. The average total sperm count was in the normal range of dogs of that size^22^. However, only half of the samples had a motility > 70% and 60% had a proportion MNS > 60%, cut-off values associated with higher reproductive performance^3,25^. The Bernese mountain dog suffers from several inheritable diseases and overall low reproductive performance. Our results suggest low semen quality to play a role in the low pregnancy results observed in the Swedish population^1^. Inbreeding and a limited genetic pool in the breed^26^, suggest that genetic factors might contribute to the observed phenotype with overall low semen quality. Even though this may influence the frequency of observed traits, it is less likely that associations between semen quality and endocrine markers are affected by genetic factors.

There was an association between semen quality and age. Increasing age was associated with a decreased proportion of MNS, decreased motility and an increased proportion of proximal droplets. The decreased proportion of MNS, driven by the increase of spermatozoa with proximal droplets, is in agreement with a previous study on various breeds^22^ and in populations consisting of single breeds^27,28^. The result is expected, since degenerative processes associated with age may disturb spermatogenesis and sperm maturation, and spermatozoa with proximal droplets are considered immature^29,30^. However, our observation of decreased motility with age is not consistent with previous studies^22,27^. In humans age has been described to correlate with both a decreased proportion of normal spermatozoa and decreased motility^20,31^.

Testicular consistency correlated with several semen parameters but was not significantly associated to age. Abnormal testicular consistency may indicate degenerative disorders, neoplasia or inflammatory disease^32,33^. The most common finding was soft testicular tissue, typically associated with degenerative conditions^32^. Testicular degeneration is associated with a high frequency of abnormal spermatozoa and may in advanced cases lead to azoospermia^32^. Even though the correlation between testicular tissue consistency and semen parameters is expected, it emphasizes the important information that can be gained from a clinical examination.

The information on semen quality in obese or overweight dogs is scarce. However, in men there are evidence of decreased sperm quality with increasing weight and obesity. Specifically, obesity is correlated to low sperm concentration, decreased sperm motility and increased DNA fragmentation^34^. In the present study we observed a correlation between increased BCS and decreased proportion of tail defects. Because of the low proportion of overweight dogs and no obese dogs included in the study, this result should be interpreted with care.

A subset of dogs mated a bitch within two months of sampling. Comparing semen parameters and fertility is challenging, as reduced semen quality to some extent can be counteracted by optimal breeding planning. Even so, semen quality clearly affects the reproductive performance of the male stud dog and there are studies suggesting reduced semen quality to affect litter size^35^ and a higher risk of failure of insemination using reduced quality semen (e.g. ^22,36,37^.).

Only few studies have examined factors related to fertility in the dog (e.g. ^22,27,28,35^,). In the present study with the small sample of dogs with recent matings showed no significant difference in semen quality was seen between the dogs achieving a pregnancy and those that did not, most likely explained by the sample size (n = 19). Further, it is possible that the semen quality differed between the day of sampling and the day of mating. Even so, it is noteworthy that despite one dog was presenting with a high number of proximal droplets and tail defects, having only 18.5% MNS, but displaying normal sperm count and motility the day of the sampling, he successfully achieved a pregnancy. In previous studies, > 60% MNS have been associated with higher reproductive performance^3,25^). This emphasises the complex nature of fertility traits. Some cases of subfertility may be successfully addressed through strategic breeding approaches^15^. This involves exclusively mating the male with the bitch during the optimal fertilization window (predicted using progesterone), thereby reducing the necessity for the spermatozoa to endure longer periods before fertilizing susceptible oocytes.

In canine theriogenology, ALP is regularly analysed to differ between obstructive and non-obstructive azoospermia^23,24^. Consequently, in dogs with spermatozoa in the ejaculate, ALP is generally not analysed. Interestingly, we found a correlation between ALP levels and total sperm count. This could possibly be explained by increased ALP activity in the epididymidis/seminiferous tubuli and further, increased production of spermatozoa. A limitation of our study is that the collection of the third fraction of the ejaculate was not standardized, leading to a variation in volume of the ejaculates. However, after correcting for ejaculate volume, the correlation persisted (data not shown). Further research with other experimental designs are needed to unravel the mechanism and investigate the relationship between sperm count and ALP further.

At puberty, the Sertoli-cell redirects the production of AMH from the bloodstream to the seminiferous tubuli, leading to a reduction in serum AMH seen at puberty in males and explaining why AMH is measurable in seminal plasma in adult men^38^, stallion^39^ and donkeys^40^. As AMH could not be detected in seminal plasma in any sample, it raises the question if dogs differ from other species investigated, and if the redirection present in other species is less pronounced in the dog. Increasing serum AMH concentrations correlated with decreasing total sperm count and increasing head defects. In theory, the spermatogenic output is determined by the total capacity of the Sertoli-cells in the testes, and it is possible that degeneration of the Sertoli cells, leading to increased AMH production, is associated to a decreased spermatogenic output. The association between AMH and total sperm count has not been previously reported in dogs. A previous study could not find a similar association^10^. The discrepancy could possibly be explained by the different dog samples, where Domain et al., (2022) included several dog breeds while the current study have only included one breed thus being able to reduce the effect of dog size on total sperm count^22^. In humans, a correlation between seminal plasma AMH and semen parameters including total sperm count has been reported^41^ while no correlation has been reported between AMH measured in serum and total sperm count^42^. AMH is higher in seminal plasma compared to serum in men^41^. The redirection of AMH to seminal plasma may suggest stronger associations between seminal plasma concentrations and semen parameters in men. In dogs, where we do not appear to have a similar redirection, associations between serum levels and semen quality may be stronger. This can be an advantage when continuing AMH research in dogs.

There was no general trend of decreasing semen quality with increasing serum AMH concentration and a cut-off value for AMH could not predict motility or MNS at a high accuracy. However, the highest serum AMH concentrations in our samples, were associated with a severe decline in sperm parameters, specifically total sperm count, motility and MNS. This suggests that instead of a linear relationship, the correlation between Sertoli cell function and semen parameters is more complex. For example, an increasing amount of normal Sertoli cells (and thus, increased production of AMH) is associated with increased spermatogenic output^43^. On the other hand, with abnormal Sertoli cell development, such as in cases of Sertoli cell tumours, the AMH production by the abnormal cells are elevated resulting in an increase of AMH above normal concentrations in blood^11^. There was an association between high AMH concentration and low MNS, but not with specific sperm defects. This further suggest that abnormal Sertoli cell function is important to several aspects of sperm morphology. However, due to few samples with high AMH, this needs to be further investigated. The monotone relationship between increasing AMH and spermatozoal head defects suggests that the Sertoli cell function is especially important for this part of spermatogenesis. This association was driven by the observations of high AMH concentration (> 10 ng/ml).

There was no linear relationship between AMH and age. However, the relationship between serum AMH concentration and dog age was not monotone, but rather suggested a U-shape association curve between age and AMH. This could not be confirmed due to our relatively low sample sizes in young (< 2 years) and old (> 8 years) dogs. Our results are in agreement with studies on the human population, in which an increase in AMH has been observed in elderly men^44^, while no obvious decrease has been observed in the adult population^45^. It is possible that similar trends are present in the dog, with an increase of AMH in elderly dogs due to age-related degeneration of the testicular tissue. Our results are interesting as Domain et al. (2022) recently reported an increase of blood AMH with increasing dog age in a population of dogs of different breeds^10^. The different observations between the studies may be a result of the different sample set, where Domain et al., (2022) included several dog breeds (and thus a variation in biological age) but excluded abnormal ejaculates such as those with blood in the third fractions and ejaculates from dogs with any sign of disease. Further research could help elucidate mechanisms involved in Sertoli cell function in dogs throughout the entire lifespan.

Inhibin B is one of the most important regulatory hormones involved in steroidogenesis. It suppresses FSH release, but also modulates other biological processes^14,46^. In men, inhibin B concentrations correlate with fertility and several sperm variables, with potential as a marker for Sertoli cell function and indirectly spermatogenesis^14^. However, there are species differences and in rats, inhibin B only reflect major spermatogenic alterations^47^. Currently, there are scarce data on inhibin B and semen quality in dogs. Our data suggest a relationship between total sperm count and inhibin, as for AMH, but more data is needed to evaluate the possible usefulness of Inhibin B as a marker for Sertoli cell function and indirectly spermatogenesis in the dog.

The dogs that presented with the lowest serum testosterone concentrations all showed poor semen quality (either low total sperm count or low MNS). We could also find a decrease in testosterone and FAI with age in our samples, in agreement to previously reported^6^. These observations are expected, as testosterone correlates with spermatogenesis and decreases with age in both men^48^ and dogs^6^. It is interesting that the correlation with age could be observed even though we were not able to standardize the sampling in regards of time of the day, as testosterone concentrations fluctuate.

In contrast to testosterone, INSL-3 reflects more chronic differentiation status of the Leydig cells^8^, and could potentially serve as a more consistent marker for Leydig cell function. For example, INSL-3 expression is increased after deslorelin-induced testicular degeneration^49^ and serves as a biomarker for Leydig cell dysfunction in men with infertility^50^. To the authors’ knowledge, this is the first time INSL-3 concentrations are presented in dogs in correlation to semen quality, and we could not observe any relationships, as was also the case between INSL-3 and age. In contrast, in men INSL-3 concentrations decrease with age^48^. Possibly, decreased sperm quality in the dog is more dependent on other factors than Leydig cell function.

Elevated CPSE concentrations (> 90 ng/ml) are associated with increased prostatic size^51^, commonly associated with prostatic hyperplasia (PH). In our studied population, a high proportion of the samples had concentrations of CPSE consistent with PH. We were not able to follow the dogs with ultrasonography. This would have been necessary to confirm if early stages of prostatic enlargement or PH was present in the vast majority of dogs or if CPSE concentrations using the current method are overestimated in large dog breeds in general or in the Bernese mountain dog specifically.

Despite the generally high prevalence of suspected PH, there was an increased prevalence of dogs with elevated CPSE concentrations in older dogs. This is an expected finding, as prostatic disease increases with age in dogs, along with increased prevalence of testicular degeneration and tumours^52^. Prostatic disease such as PH has also been associated with impaired semen quality in dogs, both decreased motility, total sperm count along with decreased proportion of MNS have been observed^53^. We observed a tendency towards a decreased motility in dogs with high CPSE, but no association with other semen parameters and CPSE.

To investigate if an autoimmune reaction could be a factor behind impaired semen quality in dogs, as previously reported for azoospermia^16^, the presence of heterophilic antibodies was investigated. The results do not support autoimmune disease to be an important factor behind impaired semen quality in our sample set. More specific markers for autoimmune reactions and possible relation to semen quality in dogs could be further studied.

Our study has limitations. To be able to recruit a sufficient number of individuals of the breed, the samples were collected by many different veterinary clinicians across the country. Even though the morphology was assessed at the same laboratory, transported samples prevented more advanced assessment of semen quality and kinematic analyses. Further, even though a relative homogenous pool of dogs have many advantages, the lack of very young (< 2 years) and very old (> 10 years) dogs limited the evaluation of non-linear fluctuations across the life of the dog.

## Conclusion

This study is the first to present data on several markers for Sertoli and Leydig cell function along with data on dog variables and correlations to semen quality using a sample set of Bernese mountain dogs, which reduces variations between dogs. Sperm motility and morphology decreased with age. More importantly, abnormal testicular palpation was associated with several semen parameters highlighting the importance and the value of the clinical examination. ALP in seminal plasma, which has previously mainly been used to differ obstructive from non-obstructive azoospermia, showed a linear relationship with total sperm count. Further studies on this relationship are warranted.

AMH was not detected in seminal plasma, in contrast to the situation in other species where redirection to seminal plasma occur along with sexual maturation. This proposes dogs to be an interesting species for further research on mechanisms of AMH. AMH was associated with several semen parameters and that AMH, and the Sertoli cell function, is highly important for the maturation of spermatozoa. While some parameters show a linear correlation, the relationship seems to be complex.

In summary, our results contribute to the understanding of factors associated with semen quality in dogs and raises new hypotheses related to especially Sertoli cell function in dogs.

## Methods

### Dog recruitment, questionnaire ethical considerations

During March to October 2020, privately owned Bernese Mountain Dogs (BMDs, n = 67) were recruited to the study from small animal veterinary clinics across Sweden. Owners were informed of the study and signed a written informed consent. Data was handled according to the general data protection regulation, GDPR, and institutional guidelines. Ethical permission was obtained through the regional animal ethical committee (Uppsala animal ethics committee, 5.8.18–17,395/2018).

Prior to sample collection, owners filled in a short questionnaire (available in Supplementary information, SI, Table S1). The questionnaire contained open and closed questions regarding medical history, medications and breeding history. Breeding history details included if the dog was previously used in breeding, if he was planned to be used in the future or if he was never used in breeding. For dogs previously used in breeding, the number of matings and results of each mating were recorded. If applicable, the date of the mating either before or after the semen sampling was noted. For dogs that mated a bitch during the period two months before to two months after sampling, owners were encouraged to send in information regarding pregnancy results.

### Clinical examination and sample collection

All dogs underwent clinical examination including determination of body condition score (BCS, scale 0–9)^54^ and weight, measurement (diameter, height, width) and palpation (normal/abnormal consistency) of the testicles and rectal palpation of the prostate.

Semen samples were collected through manual stimulation, when possible in the presence of a teaser bitch in oestrus, all three fractions were collected and the volume of the ejaculate noted. For one of the samples, semen was collected without the presence of a teaser bitch in oestrus. This dog was previously used in breeding and sampling was uncomplicated, see further details under section “Statistical analysis”. Blood was collected in tubes without additive from the cephalic vein. Blood and seminal plasma were centrifuged at 1300 × g for 10 min and the supernatants were then stored in aliquots at − 80 °C until analysed.

### Semen analysis

Volume and colour of the ejaculate were recorded. Sperm motility (%) was subjectively evaluated under a phase-contrast microscope (100 × , 200 ×). Fresh semen sample, smears and fixed aliquot in buffered formalin were sent for morphological evaluation at the semen laboratory at the semen laboratory (Swedish University of Agricultural Sciences, Department of Clinical Sciences), semen was also centrifuged at 1300 × g for 10 min and the supernatants (the seminal plasma) were then stored in aliquots at − 80 °C until analysed. The same two technicians working at the semen laboratory evaluated the semen samples. Total sperm count was calculated using the volume multiplied with the concentration calculated using a Bürker chamber. Sperm morphology was evaluated using standard procedures in wet preparations of semen fixed in buffered formalin and in air-dried smears stained with carbolfuchsin–eosin. The proportion of morphologically normal spermatozoa (MNS) and the proportion of each defect (abnormal head/midpiece/tail, presence of proximal droplets) were recorded.

### Sample processing, analytical methods

Target analytes were analysed in blood serum and/or in seminal plasma. Analyses were performed at the Department of Clinical Sciences and at the Clinical pathology laboratory at the University animal hospital at the Swedish University of Agricultural Sciences, Uppsala, Sweden.

ALP was quantified in seminal plasma from all dogs using an Architect c4000 (Abbott Laboratories). AMH was analysed in serum (n = 65) and seminal plasma (n = 16) using a sandwich ELISA developed for human serum but widely used for dogs (AMH Gen II ELISA, Beckman Coulter ^55^,) according to manufacturer’s instructions. Serum analyses of inhibin B were made with a canine ELISA (AnshLabs), of sex-hormone binding globulin, SHBG, using a canine SHBG ELISA (MyBioSource), and of INSL-3 using a quantitative sandwich ELISA (MyBioSource). Samples with concentrations above the detection range were diluted and analysed again.

Total serum-testosterone was analysed using Immulite 2000 (Siemens Healthcare Diagnostics, Elangen, Germany). CPSE was analysed in serum using Speed™ CPSE in clinic immunoassay reader (Virbac) according to manufacturer’s instruction.

Heterophilic antibodies were analysed using a modification of an ELISA developed in house^18^. In brief, plates were coated with 2 µg/ml mouse IgG over night at 4 °C. Samples and controls (50 µl) were added, incubated for 90 min and washed 4 times. Plates were then incubated for 90 min with a HRP-conjugated mouse anti-human carcinoembryonic antigen (CEA), washed and incubated with TMB where after the reaction was stopped and the optical density (OD) read at 450 nm. Chicken anti-mouse IgG diluted 1:1600 was used as positive control and a previously analysed negative dog as negative control. The cut-off was set for each plate by measuring the OD of 20 replicates of PBS and calculating mean + 4 standard deviations (SD).

### Data pre-processing

Values below limit of detection (LOD) were replaced by the lowest detected value divided by two. Dogs with two normal testicles by palpation were considered normal while one or two abnormal testicles were considered as abnormal testicles by palpation. Testicle diameter was the combined diameter of both testes. Testicle volume was estimated as V = (diameter*height*width*π)/6 and the volume of both testes added together. Eight dogs were used to validate the measurements; three repeats by 2–3 persons independent of each other or three independent repeats by the same person.

Simple- and double tail defects and coiled tails were summed to the variable “tail defects”. Similarly, “head defects” was created as the summation of acrosome and head defects. Free testosterone was estimated using the Free androgen index (FAI), calculated as the ratio between testosterone and SHBG. The prostate could be reached in around half of the dogs, allowing evaluation of the caudal pole of the prostate. Therefore, prostatic palpation was excluded from variables examined. Instead, CPSE was included as a proxy for prostatic size. A cut-off for increased prostatic size was set to CPSE > 90 ng/mL, corresponding to a prostate that is 2.5 times larger than that of a 4-year-old dog of the same weight^51^. A subset of the samples was created of 19 dogs that mated a bitch recently, defined as within two months of sample collection.

### Statistical analyses

RStudio for R (Ri386, 4.0.5)^56^ was used for statistical analyses. Dogs with azoospermia were excluded (n = 2) from the investigation of correlation of sperm parameters with other biomarkers, and one dog with azoospermia in an incomplete ejaculate (ALP < 5000 U) was excluded from the investigation of the outcome “total sperm count”. One sample was collected without a teaser bitch but with a complete ejaculate (ALP > 5000 U). The dog displayed good semen quality (> 70% motility, > 60% MNS) but with total sperm count below average in the study population. As the presence of a teaser bitch may affect the total sperm count of the ejaculate, the robustness of our findings were tested by excluding the dog from analyses with the outcome total sperm count. The results did not differ and the results with the dog included are presented.

Normal distribution of the biomarkers could not be assumed after investigation using qqplots (ggpubr package in R) and Shapiro-Wilks test. Spearman’s correlation was used to investigate the correlation between ALP and biomarkers for Sertoli and Leydig-cell function (AMH, inhibin B, FAI, total testosterone, INSL-3), semen quality (proportion morphologically normal spermatozoa (MNS) and specific sperm defects, motile spermatozoa (motility) and total sperm count) and dog variables (age, BCS).

A monotone relationship was not sufficient to describe the relationship between AMH and semen parameters. Therefore, in addition, AMH was investigated as a binary parameter with cut-off 10 ng/mL as described in adult dogs with normal testicles assessed by palpation ^11^, The association between the binary variable CPSE (> 90 ng/mL/ ≤ 90 ng/mL) and the new binary variable AMH (> 10 ng/mL and ≤ 10 ng/mL) to semen quality (MNS, specific sperm defects, motility and total sperm count) and dog variables (age, BCS) was investigated using Wilcoxon-Mann–Whitney test. To investigate if AMH could predict good semen quality (> 70 motility, > 60 MNS, alone or in combination), cutpointr package in R was used.

To test the robustness of the findings, a subset of the data was created excluding all dogs (n = 8) who received medication for any cause < 6 months of sampling. The results were consistent and hence, only the results from the full dataset are presented.

Descriptive data is presented as median and interquartile range (IQR) if not stated otherwise. A *p-*value of < 0.05 was considered significant.

### Supplementary Information

Supplementary Tables.

## Data Availability

The data underlying this article is available in the article and in its online supplementary material (Table S1-S7).

## References

[1] Axner E, Rasmus LS, Melangen T (2022). Factors affecting reproductive performance in the Swedish Bernese mountain dog. Acta Vet. Scand..

[2] Dahlbom M, Andersson M, Juga J, Alanko M (1997). Fertility parameters in male Irish wolfhounds: A two-year follow-up study. J. Small Anim. Pract..

[3] Johnston SD (1991). Performing a complete canine semen evaluation in a small animal hospital. Vet. Clin. N. Am. Small Anim. Pract..

[4] Oettlé EE (1993). Sperm morphology and fertility in the dog. J. Reprod. Fertil. Suppl..

[5] Almeida S, Rato L, Sousa M, Alves MG, Oliveira PF (2017). Fertility and sperm quality in the aging male. Curr. Pharm. Des..

[6] Holst BS, Nilsson S (2023). Age, weight and circulating concentrations of total testosterone are associated with the relative prostatic size in adult intact male dogs. Theriogenology.

[7] Laurent MR, Hammond GL, Blokland M, Jardí F, Antonio L, Dubois V, Khalil R, Sterk SS, Gielen E, Decallonne B, Carmeliet G, Kaufman JM (2016). Sex hormone-binding globulin regulation of androgen bioactivity in vivo: validation of the free hormone hypothesis. Sci. Rep..

[8] Ivell R, Wade JD, Anand-Ivell R (2013). INSL3 as a biomarker of Leydig cell functionality. Biol. Reprod..

[9] Hirobe S, He WW, Lee MM, Donahoe PK (1992). Mullerian inhibiting substance messenger ribonucleic acid expression in granulosa and Sertoli cells coincides with their mitotic activity. Endocrinology.

[10] Domain G, Buczkowska J, Kalak P, Wydooghe E, Banchi P, Pascottini OB, Nizanski W, Van Soom A (2022). Serum anti-mullerian hormone: A potential semen quality biomarker in stud dogs. Animals (Basel).

[11] Holst BS, Dreimanis U (2015). Anti-mullerian hormone: A potentially useful biomarker for the diagnosis of canine Sertoli cell tumours. BMC Vet. Res..

[12] Ano H, Hidaka Y, Katamoto H (2014). Evaluation of anti-Müllerian hormone in a dog with a Sertoli cell tumour. Vet. Dermatol..

[13] Hornakova L, Vrbovska T, Pavl'ak M, Valencakova-Agyagosova A, Halo M, Hajurka J (2017). The evaluation of blood concentrations of testosterone, 17β-oestradiol and anti-Mu¨llerian hormone in dogs with cryptorchidism and testicular tumours. Pol. J. Vet. Sci..

[14] Makanji Y, Zhu J, Mishra R, Holmquist C, Wong WP, Schwartz NB, Mayo KE, Woodruff TK (2014). Inhibin at 90: From discovery to clinical application, a historical review. Endocr. Rev..

[15] Wallace MS (1992). Infertility in the male dog. Probl. Vet. Med..

[16] Goericke-Pesch S, Reifarth L, Behrens Mathiesen C, Schuler G, Umbach AK, Korber H (2022). Chronic immune-mediated orchitis is the major cause of acquired non-obstructive Azoospermia in dogs. Front. Vet. Sci..

[17] Gehin JE, Klaasen RA, Norli ES, Warren DJ, Syversen SW, Goll GL, Bjøro T, Kvien TK, Mjaavatten MD, Bolstad N (2021). Rheumatoid factor and falsely elevated results in commercial immunoassays: Data from an early arthritis cohort. Rheumatol. Int..

[18] Bergman D, Larsson A, Hansson-Hamlin H, Svensson A, Holst BS (2018). Prevalence of interfering antibodies in dogs and cats evaluated using a species-independent assay. Vet. Clin. Pathol..

[19] Bergman D, Larsson A, Hansson-Hamlin H, Åhlén E, Holst BS (2019). Characterization of canine anti-mouse antibodies highlights that multiple strategies are needed to combat immunoassay interference. Sci. Rep..

[20] Sloter E, Schmid TE, Marchetti F, Eskenazi B, Nath J, Wyrobek AJ (2006). Quantitative effects of male age on sperm motion. Hum. Reprod..

[21] Goericke-Pesch S, Failing K (2013). Retrospective analysis of canine semen evaluations with special emphasis on the use of the hypoosmotic swelling (HOS) test and acrosomal evaluation using Spermac((R)). Reprod. Domest. Anim..

[22] Tesi M, Sabatini C, Vannozzi I, Di Petta G, Panzani D, Camillo F, Rota A (2018). Variables affecting semen quality and its relation to fertility in the dog: A retrospective study. Theriogenology.

[23] Freshman, J., Amann, R., Bowen, R., Soderberg, S. & Olson, P. Clinical evaluation of infertility in dogs. The Compendium on continuing education for the practicing veterinarian (USA) (1988).

[24] Johnston, S. D., Kustritz, M. V. R., & Olson, P. S. Canine and feline theriogenology. *Saunders* (2001).

[25] Oettlé EE (1993). Sperm morphology and fertility in the dog. J. Reprod. Fertil..

[26] Letko A, Hédan B, Snell A, Harris AC, Jagannathan V, Andersson G, Holst BS, Ostrander EA, Quignon P, André C, Leeb T (2023). Genomic diversity and runs of homozygosity in bernese mountain dogs. Genes.

[27] Hesser A, Darr C, Gonzales K, Power H, Scanlan T, Thompson J, Love C, Christensen B, Meyers S (2017). Semen evaluation and fertility assessment in a purebred dog breeding facility. Theriogenology.

[28] Foutouhi A, Hesser A, de la Fuente A, Bulkeley E, Dini P, Meyers S (2023). Sperm parameters in the Great Dane: Influence of age on semen quality. Theriogenology.

[29] Angrimani DS, Lucio CF, Veiga GA, Silva LC, Regazzi FM, Nichi M, Vannucchi CI (2014). Sperm maturation in dogs: sperm profile and enzymatic antioxidant status in ejaculated and epididymal spermatozoa. Andrologia.

[30] Briz MD, Bonet S, Pinart B, Egozcue J, Camps R (1995). Comparative study of boar sperm coming from the caput, corpus, and cauda regions of the epididymis. J. Androl..

[31] Ulubay M, Bahaettin Ulu M, Akdeniz E (2022). The effect of aging on semen parameters in normozoospermic men: A cross-sectional study. Int. J. Reprod. Biomed..

[32] Ortega-Pacheco A, Rodriguez-Buenfil JC, Segura-Correa JC, Bolio-Gonzalez ME, Jimenez-Coello M, Linde FC (2006). Pathological conditions of the reproductive organs of male stray dogs in the tropics: Prevalence, risk factors, morphological findings and testosterone concentrations. Reprod. Domest. Anim..

[33] Grieco V, Riccardi E, Greppi GF, Teruzzi F, Iermano V, Finazzi M (2008). Canine testicular tumours: A study on 232 dogs. J. Comp. Pathol..

[34] Kay, V. & Martins da Silva, S. Male obesity—Impact on semen quality. Obes. Gynecol. 119–126 (2020).

[35] Linde-Forsberg C, Forsberg M (1989). Fertility in dogs in relation to semen quality and the time and site of insemination with fresh and frozen semen. J. Reprod. Fertil. Suppl..

[36] Linde-Forsberg C, Forsberg M (1993). Results of 527 controlled artificial inseminations in dogs. J. Reprod. Fertil. Suppl..

[37] Linde-Forsberg C, Ström Holst B, Govette G (1999). Comparison of fertility data from vaginal vs intrauterine insemination of frozen-thawed dog semen: A retrospective study. Theriogenology.

[38] Li J, Hu T, Wang Y, Fu Y, Wang F, Hu R (2021). Development a nomogram to predict fertilisation rate of infertile males with borderline semen by using semen parameters combined with AMH and INHB. Andrologia.

[39] Claes A, Ball BA, Almeida J, Corbin CJ, Conley AJ (2013). Serum anti-Müllerian hormone concentrations in stallions: Developmental changes, seasonal variation, and differences between intact stallions, cryptorchid stallions, and geldings. Theriogenology.

[40] Holst BS, Panzani D, Camillo F, Svensson A, Rota A (2020). Anti-Mullerian hormone (AMH) concentrations are maximal at puberty in male donkeys and secretion is redirected from the blood stream to seminal plasma. Anim. Reprod. Sci..

[41] Andersen JM, Herning H, Witczak O, Haugen TB (2016). Anti-Mullerian hormone in seminal plasma and serum: Association with sperm count and sperm motility. Hum. Reprod..

[42] Aksglaede L, Olesen IA, Carlsen E, Petersen JH, Juul A, Jorgensen N (2018). Serum concentration of anti-Mullerian hormone is not associated with semen quality. Andrology.

[43] Verhoeven G (1999). Spermatogenesis and spermatogenic control: a state of the art. Verh K Acad Geneeskd Belg.

[44] Chong YH, Dennis NA, Connolly MJ, Teh R, Jones GT, van Rij AM, Farrand S, Campbell AJ, McLennan IS (2013). Elderly men have low levels of anti-Mullerian hormone and inhibin B, but with high interpersonal variation: a cross-sectional study of the sertoli cell hormones in 615 community-dwelling men. PLoS ONE.

[45] Aksglaede L, Sorensen K, Boas M, Mouritsen A, Hagen CP, Jensen RB, Petersen JH, Linneberg A, Andersson AM, Main KM, Skakkebaek NE, Juul A (2010). Changes in anti-Mullerian hormone (AMH) throughout the life span: A population-based study of 1027 healthy males from birth (cord blood) to the age of 69 years. J. Clin. Endocrinol. Metab..

[46] Kong X, Ye Z, Chen Y, Zhao H, Tu J, Meng T, Xiong C, Li H, Gong Y, Zheng L, Cheng B, Zhang Z (2021). Clinical application value of Inhibin B alone or in combination with other hormone indicators in subfertile men with different spermatogenesis status: A study of 324 Chinese men. J. Clin. Lab. Anal..

[47] Pfaff T, Rhodes J, Bergmann M, Weinbauer GF (2013). Inhibin B as a marker of sertoli cell damage and spermatogenic disturbance in the rat. Birth Defects Res. B Dev. Reprod. Toxicol..

[48] Anand-Ivell R, Wohlgemuth J, Haren MT, Hope PJ, Hatzinikolas G, Wittert G, Ivell R (2006). Peripheral INSL3 concentrations decline with age in a large population of Australian men. Int. J. Androl..

[49] Balogh O, Somoskoi B, Kollar E, Kowalewski MP, Gram A, Reichler IM, Klein R, Kawate N, Mester L, Walter B, Muller L (2021). Anti-Mullerian hormone, testosterone, and insulin-like peptide 3 as biomarkers of Sertoli and Leydig cell function during deslorelin-induced testicular downregulation in the dog. Theriogenology.

[50] Foresta C, Bettella A, Vinanzi C, Dabrilli P, Meriggiola MC, Garolla A, Ferlin A (2004). A novel circulating hormone of testis origin in humans. J. Clin. Endocrinol. Metab..

[51] Holst BS, Holmroos E, Friling L, Hanas S, Langborg LM, Franko MA, Hansson K (2017). The association between the serum concentration of canine prostate specific esterase (CPSE) and the size of the canine prostate. Theriogenology.

[52] Peters MA, de Rooij DG, Teerds KJ, van Der Gaag I, van Sluijs FJ (2000). Spermatogenesis and testicular tumours in ageing dogs. J. Reprod. Fertil..

[53] Angrimani DSR, Brito MM, Rui BR, Nichi M, Vannucchi CI (2020). Reproductive and endocrinological effects of Benign Prostatic Hyperplasia and finasteride therapy in dogs. Sci. Rep..

[54] Laflamme D (1997). Development and validation of a body condition score system for dogs. Canine Pract..

[55] Holst B (2017). Diagnostic possibilities from a serum sample—Clinical value of new methods within small animal reproduction, with focus on anti-Müllerian hormone. Reprod. Domest. Anim..

[56] RCoreTeam. R: A language and environment for statistical computing. In: R Foundation for Statistical Computing (2021).

