# Supplementary material for: Endocrine and dog factors associated with semen quality
Source: Sci Rep. 2024 Jan 6;14:718. doi: 10.1038/s41598-024-51242-0 (PMC10771459; doi:10.1038/s41598-024-51242-0)
Supplement: Supplementary file 1 — Supplementary Tables. [file 41598_2024_51242_MOESM1_ESM.pdf]

Supplementary information

Factors associated to semen quality in the dog

Ida Hallberg,[a,b,*] Hannah Olsson,[a] Angus Lau,[a] Stina Wallander,[a] Anna Snell,[a] Daniel Bergman,[a,c] Bodil Ström Holst[a]

[a]Department of Clinical Sciences, Division of Reproduction, The Centre for Reproductive Biology in Uppsala, Swedish University of Agricultural Sciences, SE-750 07, Uppsala, Sweden

[b]Department of Biomedical Science and Veterinary Public Health, Swedish University of Agricultural Sciences, SE-750 07, Uppsala

[c]Department of Laboratory Medicine, Karolinska Institute, SE-141 86, Stockholm, Sweden

*Corresponding author: Ida Hallberg
Swedish University of Agricultural Sciences, SE-750 07, Uppsala, Sweden
+4618671641, Ida.hallberg@slu.se

Table S1. Questionnaire 2
Table S2. Coefficient of variance 3
Table S3. Quantification of anti-müllerian hormone (AMH) in dog seminal plasma 4
Table S4 Correlation between ALP and semen quality 4
Table S5. Semen quality in dogs positive for heterofilic antibodies 5
Table S6. Data frame 6

Table S1. Questionnaire

**Name (may be abbreviated)**	**Reg. Nb:**
**ID:**	**Born:**
**Breeding history:**<br>☐Never used in breeding<br>☐Planned to be used in breeding<br>☐Have been used in breeding:<br>Matings, specify amount: _______________<br>Litters, specify amount: _______________<br>Sired puppies, specify amount: _______________	**Treated with hormones (such as chemical castration or other):**<br>☐Yes,<br>date: _______________<br>details: _______________<br>☐ No
**Other health questions:**<br>Problems urinating (stranguria, blood in urine, dripping):<br>☐Yes<br>☐ No<br>Problems with defecating:<br>☐Yes<br>☐ No<br>Other: ____	**Treatment history:**<br>☐Never prescribed medications<br>☐Treated with tick-profylaxis during the last year<br>Type: _______________ Date: ______________<br>☐Other medications<br>Type: _______________ Date: ______________
Other information (for example specific breeding history details or medication details or anything else you would like to mention):	

Table S2. Coefficient of variance

Table S2a: Coefficient of variance between different persons (A-C) measuring testicle diameter and volume of 8 dogs.

Dog sample	Performer ID	Calculated volume. cm^3	SD	Mean volume (cm^3)	CV (%)	Diameter (mm)	SD	Mean diameter (mm)	CV (%)
1	A	25	1.5	26	5.8	62	9.9	55	18
	B	27				48			
2	A	44	0.5	44	1.2	60	1.4	61	2.3
	B	44				62			
3	A	31	6.9	26	26.3	45	0	45	0
	B	21				45			
4	A	60	7.2	55	13	58	2.1	56.5	3.8
	B	50				55			
5	A	44	4.5	41	11	50	1.4	51	2.8
	B	37				52			
6	A	50	9.2	46	19.9	50	5.3	48	11
	B	35				42			
	C	52				52			
7	A	41	11.0	33	33.9	54	5.5	48.3	11.4
	B	37				48			
	C	20				43			
8	A	40	6.4	33	19.3	59	6.2	54	11.6
	B	28				47			
	C	30				56			

Table S2b: Coefficient of variance between measurements of the same person measuring testicles of three dogs in three different time-points

Dog sample	Calculated volume. cm^3	SD	Mean volume (cm^3)	CV (%)	Diameter (mm)	SD	Mean diameter (mm)	CV (%)
1	52	7.3	44	16.8	52	1	52	1.9
	39				51			
	41				53			
2	20	1.3	20	6.6	43	2	43	4.7
	19				45			
	22				41			
3	30	1.5	29	5	56	4.6	50.7	9.1
	28				48			
	30				48			

Table S3. Quantification of anti-müllerian hormone (AMH) in dog seminal plasma

ID	Age[a]	Volume[b]	Sperm count[c]	Motility[d]	MNS[e]	AMH_{serum}[f]	AMH_{semen}[f]
1	33	4.5	1.28	90	59.5	9.18	<0.2
2	104	3	0.15	5	10.5	16.71	<0.2
3	18	6	0.73	20	39	4.03	<0.2
4	33	4.5	1.82	50	32	2.8	<0.2
5	47	13	0.09	15	49	8.04	<0.2
6	118	3	0.22	50	20	3.89	<0.2
7	56	4	0	-	-	28.48	<0.2
8	36	8	3.02	85	47.5	5.73	<0.2
9	66	27	0.51	70	44.5	6.02	<0.2
10	65	9.5	2.07	60	83	4.29	<0.2
11	47	7	1.15	75	44	4.49	<0.2
12	78	7	1.77	70	35.5	3.79	<0.2
13	17	4	0.92	80	77.5	4.09	<0.2
14	19	1	0.32	80	79.5	6.78	<0.2
15	40	3.4	1.55	60	34.5	7.5	<0.2
16	41	2.8	1.46	70	80	6.33	<0.2

[A]Age presented in months, [b]Volume in ml, [c]sperm count x 10^9, [d]proportion of motile sperm assessed by phase contrast microscopy, [e]proportion morphologically normal sperm, [f]AMH in serum and seminal plasma in ng/ml.

Table S4 Correlation between ALP and semen quality

Table S3a: In samples with total sperm count > 300 million spermatozoa

Variable	N	Correlation	95% CI for ρ	P-Value
Age	51	-0,188	(-0,443; 0,095)	0,187
Motility	51	0,183	(-0,099; 0,439)	0,198
Sperm_total	51	0,447	(0,183; 0,652)	0,001
MNS	51	0,138	(-0,145; 0,399)	0,336
pat_head	51	-0,245	(-0,491; 0,037)	0,083
midpiece	51	0,034	(-0,244; 0,307)	0,811
tails	51	-0,106	(-0,371; 0,176)	0,460
immature	51	-0,106	(-0,371; 0,176)	0,461

Table S3b: In samples with total sperm count > 600 million spermatozoa

Variable	N	Correlation	95% CI for ρ	P-Value
Age	43	-0,154	(-0,436; 0,155)	0,323
Motility	43	0,308	(0,001; 0,562)	0,044
Sperm_total	43	0,427	(0,132; 0,653)	0,004
MNS	43	0,176	(-0,134; 0,454)	0,259
pat_head	43	-0,207	(-0,480; 0,103)	0,183
midpiece	43	0,132	(-0,177; 0,417)	0,400
tails	43	-0,132	(-0,417; 0,177)	0,399
immature	43	-0,148	(-0,431; 0,161)	0,344

Table S5. Sensitivity and specificity for using AMH as predictor of semen quality

Endpoint	Optimal cut-point	Sensitivity	Specificity
Motility > 70%, MNS > 60%	5.14	0.60	0.56
Motility > 70%	5.44	0.59	0.67
MNS > 60	5.14	0.55	0.63

Table S6. Semen quality in dogs positive for heterophilic antibodies

Sample ID	MNS (%)	Total sperm count	Motility (%)
1	<10	0,28	30
2	75	1,78	80
3	80	1,17	83
4	79	0,97	70
5	73	0,84	80
6	56	0,93	80

Table S7. Data frame

Age(m)	Breeding-details	Recent_mating	Recent_mating_result	Volume	color_cat	Motility	BCS	Weight	Testicles_dia	Sin_dia	Sin_height	Sin_width	Dx_dia	Dx_height	Dx_width	Sin_consistency	Dx_consistency	Testicles_consistency	prostate	Concentration	Sperm_total	AMH_serum	AMH_seminalplasma	testosteron	SHBG	CPSE	INSL-3	ALP_ukat	ALP_U	ALP*volume	pat_head	immature	loose_heads	acro_defects	acro_errors	vacuoles	midpiece	simple_tail	coiled_tail	double_tail	spermatogenic_binary	spermatogenic_scale	degenerate_scale	Leukocyter_scale	inhibin_mean
18	not used for breeding			2	normal		6	50	46	22	24	45	23	29	44	normal	normal	normal		0,57	0	0,79		4,5	41,14	53,57	110,88	1,8	105,88	0,21	0,00	5	2	2,00	0	0	0	4	8	3	0	0	2	0	10,58
56	used for breeding			4	normal	0	6	50	39	21	24	44	24	25	45	soft	soft	abnormal		0	0	28,48	<0,2	9,6	45,74	341,82	93,09	265,6	15623,53	62,49	0,00	0	0	0,00	0	0	0	0	0	0	0	0	0	0	6,1
99	used for breeding	Yes	No pregnancy	30	abnormal	0	5	35	50	30	40	25	20	40	20	soft	soft	abnormal		0	0	12,07		2,2	24,45	500	3,47	141,5	8323,53	249,71	0,00	0	0	0,00	0	0	0	0	0	0			0	0	2,96
45	not used for breeding			35,5	blood		4	45,5	47	44	24	44	45	25	48	normal	normal	normal		0,5	0,02	4,12		9,5	56,66	500	120,24	1,7	100	3,55	5,00	4,5	0,5	1,50	0	0,5	0,5	12,5	22,5	5	0	0	0	0	14,42
48	used for breeding	Yes	Pregnancy	0,5	normal	98	5	48,5	80	40	50	25	40	50	25	normal	normal	normal		59	0,03	2,88		12,3	40,95	500	122,03		100	0,005	4,80	15,5	0,5	0,00	0	0	2	4,5	0,5	0	1	2	0	2	3,69
137	used for breeding	No		2	abnormal	5	3	45	43	22	22	44	23	18	41	soft	slight soft	abnormal		36	0,07	10,14		5,4	34,68	500	170	0,85	4,7	0,01	8,40	34,3	4,8	2,50	0,8	8,3	2,8	1,5	10,3	9,8	0	0	0	4	4,7
115	used for breeding			8	abnormal	80	6	56	42	23	21	51	21	25	50	soft	slight soft	abnormal		11	0,09	9,51		0,4	41,62	352,51	105,25	255,8	15047,06	120,38	24,40	58	1	5,50	0	0,5	8	1	16	1,5	0	0	3	3	4,42
47	not used for breeding			13	normal	15	5	54,5	55	25	35	58	30	32	60	normal	soft	abnormal		7	0,09	8,04	<0,2	4,5	57,91	500	75,82	28,9	1700	22,1	10,00	15,5	0,5	1,50	0	0	3,5	10,5	13	4	1	1	0	4	9,33
83	used for breeding			8	blood	80	6	55	45	24	24	50	25	26	49	normal		normal		11	0,09	4,18		5,7	49,62	500	86,89	17,2	1011,76	8,09	6,00	25,5	1	0,00	0	1	2	1	1	0	0	0	0	4	9,38
33	planned for breeding			6	abnormal	80	5	47	40	24	28	44	21	24	45	normal	normal	normal	normal	24	0,14	4,71		0,4	26,29	64,46	323,34	48,4	2847,06	17,08	3,00	7,8	0,3	1,50	0	0,3	1,5	9,8	8,5	4,3	1	2	0	2	14,32
104	used for breeding			3	normal	5	5	57	43	31	35	51	21	28	42	normal	soft	abnormal		50	0,15	16,71	<0,2	7,1	16,52	500	259,12	9087	534529,41	1603,59	13,20	16,5	34	1,50	0	1	3	4	18	9	0	0	0	3,5	9,33
24	used for breeding			8	normal	30	5,5	52	50	22	42	19	21	40	20	soft	slight soft	abnormal	normal	22	0,18	6,81		9,9	47,22	85,65	1163	108,2	6364,71	50,92	37,90	31,3	9,3	22,00	0	0,3	11,5	6,8	14,3	3,8	1	2	0	4	16,84
118	not used for breeding			3	abnormal	50	5	44,7	56	30	28	48	36	36	50	normal	slight hard	abnormal		74	0,22	3,89	<0,2	20,5	600	500	110,3	262,9	15464,71	46,39	10,20	52,5	4	13,00	0	1,5	2,5	0,5	9,5	2	1	2	0	4	26,63
71	not used for breeding			8	normal	70	4	47,5	63	32	28	44	32	27	40	normal	soft	abnormal		33	0,26	2,63		4,1	100,96	500	93,63	69,6	4094,12	32,75	22,60	22	4	37,50	0	2,5	3,5	10	12,5	2	0	0	0	1	61,41
19	planned for breeding			1	normal	80	5	45	80	40	40	65	40	40	65	normal	normal	normal		319	0,32	6,78	<0,2	8,2	195,14	119,67	3,47	8637	508058,82	508,06	3,20	3	0,5	4,00	0	0	1	9,5	3	0,5	0	0	0	2	24,28
20	used for breeding			3	normal	75	3	43	75	55	50	30	35	65	35	normal	normal	normal	normal	128	0,38	9,76		8,7	170,09	243,05	86,41	61	3588,24	10,76	2,80	4	0,5	4,00	0	0	2,5	29	6,5	7,5	1	2	3	4	16,94
25	not used for breeding			4	normal	80	5	50	58	26	41	60	32	28	55	normal	normal	normal	normal	102	0,41	8,03		11,9	31,36	500	38,9	33,6	1976,47	7,91	9,80	6	0,5	3,80	0	0	2	11,8	6,3	7	0	0	0	4	17,36
57	used for breeding	Yes	Pregnancy	13	normal	80	5	52	52	32	45	23	26	47	21	normal	slight soft	abnormal	normal	33	0,43	4,6		8,6	43,53	500	195,55	26,6	1564,71	20,34	5,00	0,5	1	0,00	0	1,5	1,5	4,5	7,5	2	1	2	0	3	14,63
62	used for breeding	Yes	Pregnancy	3	blood	90	7	43	60	30	30	45	30	30	45	normal	normal	normal		85	0,43	7,1		3,8	38,92	500	3,47	64	3764,71	11,29	11,60	7,8	3,8	11,80	0	0,3	6	4,3	1,8	3,3	1	1	0	3	12,84
19	planned for breeding			5	normal	75	4,5	45	70	35	60	35	35	60	35	normal	normal	normal	normal	123	0,49	3,85		7,9	36,99	82,66	159,66	286,5	16852,94	84,26	3,80	2	1	0,00	0	1	2	5	3,5	2	0	0	0	0	3,14
66	used for breeding			2	blood	70	3,5	49,6	65	35	65	65	35	65	65	normal	normal	normal		253	0,51	6,02	<0,2	16,6	335,5	500	449,52	5,5	323,53	0,65	5,80	27	3,8	10,00	0	1,3	5,3	6	5,3	1,8	1	1	0	2	11,3
78	used for breeding			3,5	blood	30	6	56	110	60	50	40	70	60	45	normal	normal	normal		149	0,52	3,28		4,3	200	500	3,47	280,2	16482,35	57,69	5,40	27	8	0,50	0	0,5	3	17	12	6,5	0	0	0	4	3,96
34	used for breeding			6	blood	50	5	49	36	19	28	46	19	22	44	soft	soft	abnormal		101	0,61	5,52		6	159,58	500	3,47	99,8	5870,59	35,22	18,50	29,5	0,3	6,00	0	0,5	3	17,3	21	2,8	0	0	0	3	7,04
91	used for breeding			8	normal	50	5	50,5	45	22	31	44	22	33	42	normal	normal	normal	slight enlarged	83	0,66	6,41		4,7	54,04	500	3,47	195,2	11482,35	91,86	9,00	8	1	1,50	0	0,5	2	25	21,5	9,5	1	1	0	3	6,83
58	used for breeding			15	blood	60	5	50,8	64	32	48	25	33	45	28	normal	normal	normal		55	0,72	5,66		11,9	50,07	500	486,63	7,1	417,65	6,26	6,00	12	3	13,00	0	0	3	15,5	1	7,5	1	2	1	2,5	13,8
18	not used for breeding			6	normal	20	5	53,7	52	25	26	47	28	22	47	normal	normal	normal		122	0,73	4,03	<0,2	5,8	22,42	149,73	3,47	188,2	11070,59	66,42	6,00	5	6	0,50	0	0,5	1	19	16	11	0	0	1	1,5	6,83
35	used for breeding	Yes	Pregnancy	10	normal	55	5	49,2	58	27	40	45	28	44	54	normal	normal	normal		76	0,76	0,77		4,1	67,27	85,5	119,94	76,8	4517,65	45,18	6,40	11	0,5	1,00	0	0	0	24	12,5	6	1	1	0	1	11,71
52	not used for breeding			8	normal	50	5	51	52	24	29	43	25	31	45	soft	slight soft	abnormal		97	0,78	5,44		4,4	59,07	137,58	3,47	144,7	8511,76	68,09	1,60	13,5	1,5	1,30	0	0,3	3	7,3	42	8,5	0	0	0	2,5	33,74
79	used for breeding	No		11	normal	80	5	49,4	40	24	20	60	21	26	58	slight soft	slight soft	abnormal		76	0,84	3,57		5,3	200	459,9	97,2	120,1	7064,71	77,71	5,60	7,5	1	2,50	0	0,5	1	11,5	1	0,5	0	0	1	3	49,91
17	used for breeding	Yes	Pregnancy	4	normal	80	5	54	56	26	28	48	22	22	48	normal	normal	normal		231	0,92	4,09	<0,2	7,1	148,17	42,84	189,4	326,9	19229,41	76,92	4,60	7	3	2,00	0	0	4,5	3	2,5	2,5	0	0	2	2	16,56
102	used for breeding	No		15	normal	80	4	48	52	25	27	50	22	25	45	normal	normal	normal	normal	62	0,93	2,72		5,9	200	500	91	80	4705,88	70,59	3,80	5,5	2	0,50	0	0,5	3	21,5	5	5	1	1	0	3	22,91
98	used for breeding	Yes	Pregnancy	1,5	normal	55	4	45	80	50	70	20	50	60	30	slight soft	soft	abnormal		622	0,93	4,8		0,4	48,6	500	270,06	336,6	19800	29,7	4,60	7,5	1	1,50	0	1	2,5	21	14	12,5	0	0	0	4	14
66	used for breeding			8,5	normal	70	4	49	62	32	31	40	30	30	50	soft	soft	abnormal	normal	114	0,97	5,81		4,6	94,95	500	93,36	133,9	7876,47	66,95	5,80	6	1	1,50	0	3	2	1,5	1,5	2,5	0	0	1	2	19,2
18	planned for breeding			5	normal	75	5	44,7	68	29	34	52	26	25	47	normal	slighly soft	abnormal	normal	201	1,01	3,45		6,3	115,06	153,54	61,41	13137	772764,71	3863,82	9,40	6,5	1	6,50	0	0,5	1,5	22,5	9,5	2,5	0	0	2	3	76,57
55	used for breeding	Yes	No pregnancy	5	normal	80	7	52,4	50	30	40	30	20	40	25	normal	normal	normal	normal	205	1,03	7,59		11,8	133,71	221,82	80,78	338,8	19929,41	99,65	5,40	4	1	1,50	0	0	3	2,5	1,5	1	0	0	0	2	7,72
37	planned for breeding			9	blood	85	5	50,5	48	21	24	46	24	25	46	normal	normal	normal	slight enlarged	121	1,09	5,09		7,5	54,5	500	3,47	102,5	6029,41	54,26	1,40	5	0,5	2,00	0	0	3	3,5	2,5	1	1	2	0	3	9,12
47	used for breeding	Yes	No pregnancy	7	normal	75	6	55	60	30	27	40	32	30	38	normal	normal	normal		164	1,15	4,49	<0,2	10,7	76,07	500	57,37	9684	569647,06	3987,53	7,80	26,5	3	1,00	0,5	0	2,5	5,5	14,5	3	1	2	0	2	18,11
25	used for breeding	Yes	Pregnancy	12,5	normal	45	4	48	45	24	25	45	24	32	42	normal	normal	normal	normal	89	1,16	5,68		4,9	133,81	239,88	90,98	99,3	5841,18	73,01	8,20	3,5	7	0,00	0	3	8,5	38	13	24			0	0	
45	planned for breeding			17,5	normal	80	6	44,3	50	25	25	50	25	25	50	normal	normal	normal	normal	67	1,17	4,39		3,6	66,39	500	6,94	108,7	6394,12	111,9	6,80	6,5	1	1,50	0	0	3	1	1,5	0,5	1	2	0	2	12,44
65	used for breeding	Yes	Pregnancy	11,5	normal	75	4	55	52	25	32	45	30	28	49	soft	normla	abnormal		105	1,21	5,14		5,3	41,59	500	3,47	154	9058,82	104,18	4,20	9	0,3	4,30	0	0,3	1,8	4	19,5	6,8	0	0	0	2	17,9
44	used for breeding			17,5	abnormal	75	5	43,5	50	24	34	46	26	36	49	normal	normal	normal	normal	67	1,21	6,69		7	32,75	500	179,97	70,8	4164,71	72,88	7,60	7,3	0,8	2,00	0	0	2	9	3	3	0	0	0	2	5,63
33	used for breeding	Yes	Pregnancy	17	normal	80	5	40	55	30	35	60	25	35	65	normal	normal	normal	normal	71	1,21	8,11		12,1	41,23	500	3,47	210,3	12370,59	210,3	6,40	1,5	2,5	2,50	0	0	3	2,5	1,5	2	1	1	0	2	9,74
65	used for breeding			7	blood	75	6	53,3	57	30	28	26	30	27	45	normal	normal	normal		176	1,23	2,58		9,9	47,03	500	128,66	13065	768529,41	5379,71	8,60	31,5	5	12,00	0	1	4	2,5	7	4	0	0	2	2	9,74

33	used for breeding	Yes	Pregnancy	4,5	normal	90	5	54	71	36	50	29	32	54	29	normal	normal	normal		285	1,28	9,18	<0,2	7,9	18,92	80,35	172,7	9390	552352,94	2485,59	6,70	5,8	0,3	0,50	0	0,3	4,3	17,3	5	4,3	0	0	1	1	15,99
21	used for breeding	Yes	Pregnancy	10	normal	80	5	51	51	32	24	47	28	25	42	normal	normal	normal		135	1,35	3,38		10,5	197,8	116,8	41,22	138,3	8135,29	81,35	8,60	16,3	1,8	1,50	0	2,3	3,3	4	9,3	3	1	1	1	1	21,56
52	used for breeding	Yes	No pregnancy	10	normal	55	5	51	53	24	38	48	26	32	54	normal	normal	normal		135	1,35	4,67		7,6	64,82	164,63	39,65	193,2	11364,71	113,65	6,00	2,5	5	3,00	0	0	2	21,5	39	17	0	0	0	1	9,33
49	used for breeding			5	abnormal	65	5	39,5	60	30	40	40	30	40	40	normal	rough	abnormal	normal	277	1,39	7,21		13	101,97	500	81,53	34,7	2041,18	10,21	7,00	11	0	8,00	0	0	1	9,5	43	18,5	1	1	0	3	12,03
19	used for breeding	Yes	No pregnancy	10	normal	50	5	46	50	20	32	48	29	25	41	slight soft	normal	abnormal	normal	142	1,42	6,76		8,5	43,26	224,77	167,55	41,4	2435,29	24,35	5,40	15	2	16,50	0,5	2,5	2,5	4	4	6	0	0	2	3	19,5
41	not used for breeding			2,8	normal	70	4,5	57	50	25	35	55	25	40	55	normal	normal	normal		521	1,46	6,33	<0,2	8,6	50,68	143,9	3,47	18030	1060588,24	2969,65	2,20	2,5	1	0,50	0	1,5	1,5	4	4,5	4,5	0	0	0	1	16,24
19	not used for breeding			10	blood	85	5	58,1	45	22	24	42	26	27	35	normal	normal	normal		151	1,51	3,79		10	51,09	45,44	34,89	224,3	13194,12	131,94	5,50	1,8	1,5	1,00	0	1	2	16,8	6,3	3	0	0	0	3	15,68
40	used for breeding			3,4	abnormal	60	5	58	55	25	30	50	25	30	45	normal	slight soft	abnormal	normal	456	1,55	7,5	<0,2	1	200	157,89	1152,33	105,3	6194,12	21,06	6,80	12,3	3	3,30	0,5	8,3	5	13	8,8	16,8	1	2	0	2	8,4
42	used for breeding	Yes	Pregnancy	10	abnormal	80	4	50	48	28	26	35	27	29	34	normal	normal	normal	normal	163	1,63	3,11		6,1	46,2	500	85,25	333,2	19600	196	3,40	8,5	1,5	0,00	0	0	1	2	1,5	1,5	1	1	3	2,5	13,59
18	not used for breeding			9	normal	85	6	42,5	61	30	30	45	30	28	48	normal	normal	normal		192	1,73	5,79		20,6	85,93	325,67	88,46	12924	760235,29	6842,12	4,20	3	2	2,50	0	1,5	1,5	1,5	3	0,5	0	0	0	2,5	112,1
78	used for breeding			7	normal	70	5	53,5	53	26	30	48	25	32	50	normal	normal	normal		253	1,77	3,79	<0,2	4,2	168,81	500	93,3	6,1	358,82	2,51	5,00	35	2	2,50	0	0,5	4	8,5	7,5	2,5	1	2	0	2	12,94
54	used for breeding	Yes	Pregnancy	11	blood	80	4	55	48	27	26	50	23	29	53	normal	normal	normal	normal	162	1,78	2,17		7	600	500	490,89	198	11647,06	128,12	4,00	9,8	0,5	0,00	0	0,5	2	4	5,5	2,8	0	0	2	3	16,63
33	not used for breeding			4,5	normal	50	4	42	40	22	31	45	21	20	50	soft	soft	abnormal		404	1,82	2,8	<0,2	10,1	84,27	154,96	431,07	66795	3929117,65	17681,03	4,00	27,5	3	0,00	0	1	4,5	9,5	15,5	8	0	0	0	2	10,57
123	used for breeding			12	normal	10	5	41,9	53	29	38	48	27	39	42	normal	normal	normal		153	1,84	16,71		16,5	279,57	500	166,98	207,3	12194,12	146,33	8,80	6	28,8	0,30	0	0,5	3,3	6,5	31,3	9,3	1	1	0	2	31,97
60	used for breeding			13	normal	85	5	45,4	55	25	25	45	27	25	48	normal	normal	normal		158	2,05	3,37		4,4	36,16	500	105,73	356,2	20952,94	272,39	3,20	6	0,5	0,50	0	0,5	4	1	3,5	0,5	1	1	0	2	14,94
65	used for breeding			9,5	normal	60	6	49,2	55	27	29	60	25	28	55	normal	normal	normal		218	2,07	4,29	<0,2	14,6	175,99	500	61,41	344,3	20252,94	192,4	6,40	2,5	1,5	0,50	0	1,5	2,5	0	3,5	1,5	1	2	0	3	25,02
53	not used for breeding			11	normal	70	5	47,8	70	27	45	55	45	65	65	soft	slight soft	abnormal	normal	191	2,1	3,05		12,1	47,12	142,81	149,39	13002	764823,53	8413,06	4,20	8	1	1,00	0,5	0,5	3,5	17	7,5	6,5	0	0	0	2	11,71
111	used for breeding			5	normal	60	6	50,3	80	40	70	45	40	70	45					432	2,16	6,74		0,4	21,66	289,9	3,47	280,6	16505,88	82,53	8,20	75,5	5	4,00	0	0	4	0	12,5	1,5	1	2	0	1	7,72
17	used for breeding	Yes	Pregnancy	10	normal	80	5	54	50	27	26	56	25	23	51	slight soft	normal	abnormal	normal	242	2,42	3,98		11,5	56,9	52,26	90,64	234	13764,71	137,65	15,40	36,3	3	7,00	0	15,5	26,3	1,5	1	2,5	1	2	2	2,5	21,12
36	used for breeding			8	normal	85	6	50,5	46	22	26	41	22	25	41	normal	normal	normal		378	3,02	5,73	<0,2	6,1	19,47	80,12	3,47	15165	892058,82	7136,47	2,40	22	1	2,00	1	0	4	22	36	24	1	1	0	1	16,52
53	used for breeding			21	blood	75	5	56	65	30	40	55	35	35	60				normal	152	3,19	5,25		7,4	43,74	500	3,47	229,6	13505,88	283,62	3,60	8,5	0,5	1,50	0	0	2	28,5	7	6	0	0	0	3	6,76
16	not used for breeding			10	normal	90		50,5	45	30	25	40	21	24	35	normal	normal	normal		365	3,65	3,54		12,9	31,36	500	3,47	9723	571941,18	5719,41	3,40	2	4	2,00	0	0	3	31,5	7,5	6	1	1	0	1	15,47
